# Competency-based education in intensive care multiprofessional training: a scoping review

**DOI:** 10.62675/2965-2774.20250385

**Published:** 2025-08-27

**Authors:** Thais Oliveira Gomes, Fernanda Berchelli Girão, Matheus Henrique Silva, Marcus Vinicius Melo de Andrade

**Affiliations:** 1 Hospital das Clínicas Universidade Federal de Minas Gerais Belo Horizonte MG Brazil Hospital das Clínicas, Universidade Federal de Minas Gerais - Belo Horizonte (MG), Brazil.; 2 Center of Biological and Health Sciences Universidade Federal de São Carlos São Carlos SP Brazil Center of Biological and Health Sciences, Universidade Federal de São Carlos - São Carlos (SP), Brazil.; 3 Universidade Federal de Minas Gerais Belo Horizonte MG Brazil Universidade Federal de Minas Gerais - Belo Horizonte (MG), Brazil.

**Keywords:** Competency-based education, Clinical competency, Graduate education, Intensive care units, Critical care

## Abstract

**Objective:**

To map the development and implementation of competency-based education in intensive care multiprofessional training on the basis of national and international literature.

**Methods:**

A scoping review was conducted with searches in six databases and gray literature.

**Results:**

The initial search identified 1,636 potentially eligible records, and 31 studies were included in the final sample. The data were grouped into three themes: development of competency-based education, implementation of competency-based education in curricula, continuing education and training programs, and student assessment. The studies were published between 2000 and 2024 and focused primarily on medical education, with a predominance of publications from North America and Europe. Heterogeneity was identified in the conceptual strategies of competency-based education, with an initial focus on skill lists evolving into core competencies, milestones, and entrustable professional activities. Benefits such as transparency in the learning process, individualized tracking, and the promotion of reflective learning were identified. Challenges to implementing effective competency-based education include the need for faculty training, resistance to change, a lack of time and resources, and the development of more robust assessment tools. The lack of studies on competency-based education in Latin America and the reduced number of studies in other health fields, such as nursing and physiotherapy, were highlighted.

**Conclusion:**

Competency-based education appears promising for training in intensive care; however, further research is needed to assess its impact on quality of care and patient safety, as well as to broaden the discussion to include diverse contexts and health fields.

## INTRODUCTION

Health education has undergone changes in recent years, with a focus on a complex and multifaceted evaluation process through the individualized monitoring of learner progress. This aims to identify the competencies required to provide safe, high-quality care that meets the current demands of health care systems.^(1)^ Competency is an individual attribute and can be defined as observable and measurable actions based on the ability to integrate knowledge, behavior, skills, values, and attitudes.^(2)^

The absence of a clear model for applying competencies within health education and assessment underscores the challenge of translating the broad and often abstract concept of competencies into practical, professional applications.^(1)^

In this context, competency-based education (CBE) has garnered the interest of the academic community since the mid-1970s and has been widely adopted in medical education since the 2000s.^(3)^Competency-based education emphasizes learning outcomes, is flexible and student centered, and breaks away from the traditional time-based training model.^(3)^ Its objective is to define and assess competency throughout a trajectory, from novice to expert, using objective performance metrics.^(3)^ This approach is fundamentally guided by four components: a competency framework based on observable behaviors; curricular components and content shaped by societal needs; a focus on the teaching–learning process centered on the student and the learning achieved; and limited reliance on time-based training and numerical goals.^(4)^

Thus, various conceptual and methodological strategies have emerged to develop evaluation systems focused on professional performance, identifying critical points in training, and translating and operationalizing the concept of CBE.^(5)^ Notable among these are milestones and entrustable professional activities (EPAs).^(5)^ Milestones are progressive descriptions of the knowledge, skills, and attitudes required for each of the competencies expected during trainee education. They outline learner progression through observable behaviors.^(4,5)^ Entrustable professional activities, on the other hand, are units of professional practice entrusted to a learner once the competencies required to perform professional practice reliably have been demonstrated.^(4,5)^

Although widely disseminated, the concept of CBE is highly variable in the literature. The lack of consensus and definitions related to CBE may hinder the advancement of discussions and its effective implementation across different professional categories and specialties in health care.^(3)^

In the context of multiprofessional training in intensive care, patients requiring complex care, such as constant support therapies and monitoring, demand a highly qualified team.^(6)^ The literature highlights that the training and ongoing education of multiprofessional teams in intensive care are essential for achieving favorable patient outcomes, including shorter hospital stays, reduced treatment times and costs, lower mortality rates, and increased patient satisfaction.^(7)^ However, the COVID-19 pandemic exacerbated a preexisting issue regarding the scarcity of human resources in intensive care and highlighted gaps in the training of these professionals.^(8)^

A European study highlighted substantial variations in medical training for intensive care, including the granting of certifications, duration, format, and evaluation methods. In Europe alone, 37 different training programs were identified, with the required minimum duration ranging from 3 to 72 months.^(9)^ Studies on the training of intensive care nurses yielded similar results regarding the heterogeneity of their education, with durations varying from 30 days to 24 months.^(10)^ An Australian study on intensive care nursing training demonstrated considerable variation in course offerings and postgraduate practice outcomes. While most programs used competency standards as a framework for course curricula, inconsistencies were identified in translating these standards into observed and expected practice outcomes.^(11)^

Competency assessment in clinical settings, which is based on objective and measurable criteria, is an essential step in implementing CBE and remains a concern for preceptors. Progress tests have been implemented in contemporary training programs.^(12)^ However, these tests assess only knowledge and fail to capture all dimensions of competency, especially the affective and psychomotor domains.^(12)^ Moreover, they do not account for the full complexity of bedside clinical judgment and decision-making, leaving unanswered a key challenge for tutors: when to entrust an activity to a trainee and how to determine if they are ready for autonomous specialized practice.^(5)^

Therefore, the discussion about multiprofessional training in intensive care within the context of CBE is both current and pressing, as there is no uniform understanding of what constitutes the appropriate outcome of multiprofessional training in intensive care beyond the minimum level required for certification. Additionally, there is a lack of clarity regarding the most effective way to evaluate students’ true progress and autonomy.

This raises the question of what competencies are expected to be demonstrated upon completion of training, reflecting the specificities of clinical practice. It emphasizes not only the process but also the outcome of training: the ability to perform independent and unsupervised professional practice in a safe and qualified manner.

To address this, a scoping review was conducted to map how competency-based education has been developed and implemented in the context of multiprofessional training in intensive care, on the basis of the national and international literature.

## METHODS

This review is based on the recommendations of the international guide Preferred Reporting Items for Systematic Reviews and Meta-Analyses extension for Scoping Reviews (PRISMA-ScR),^(13)^ with the protocol registered in the Open Science Framework (OSF) under number 10.17605/OSF.IO/NR58E. The review was conducted following the methodological guidelines of the Joanna Briggs Institute (JBI).^(14)^

### Stage 1: Identification of the research question

A bibliographic survey was conducted in June 2024 to find scoping review studies related to the themes of this research, consulting OSF, the Cochrane Library, the International Prospective Register of Systematic Reviews, and the JBI Clinical Online Network of Evidence for Care and Therapeutics. This search revealed that there were no review protocols or publications with objectives similar to those of this study.

The guiding question was defined using the population, concept, and context (PCC) strategy, which consisted of postgraduate health education (P), competency-based education (C), and intensive care (C). Thus, the following guiding question was formulated: “How has competency-based education been developed and implemented in intensive care postgraduate health education?”

### Stage 2: Identification of relevant studies

The search was conducted in August 2024 using the following databases: LILACS via the Regional Portal of BVS; MEDLINE via PubMed; Embase; Cochrane Library; Scopus; and Web of Science. The search strategy was defined by the research team and reviewed by a librarian.

Descriptors and/or synonyms found in the descriptors in health sciences (DeCS) and their corresponding medical subject headings (MeSH) were used for each search item, according to the PCC strategy: population (P): health postgraduate programs OR postgraduate medical education OR postgraduate pharmacy education OR postgraduate nursing education OR postgraduate education; concept (C): competency-based education OR clinical competence OR professional competence; and context (C): critical care OR intensive care units OR intensive care unit OR intensive care.

The gray literature was explored by consulting the thesis and dissertation repositories of universities recognized for their knowledge production on the study’s theme, both nationally and internationally (University of São Paulo, University of Groningen, and University Medical Center Utrecht). Additional sources included the ProQuest Dissertations and Theses database and the Brazilian Coordination for the Improvement of Higher Education Personnel (CAPES) thesis and dissertation repository. Additionally, the references of the selected studies were reviewed.

### Stage 3: Selection and inclusion of studies

Searches were conducted in the databases, and the identified articles were imported into the reference management software EndNote Web®. Two researchers independently and blindly selected articles by reviewing titles, abstracts, and full texts via Rayyan® software.^(15)^ All the researchers were trained in the methodology employed in the review and in the use of the software. Discrepancies were discussed in face‒to-face meetings to reach a consensus, and any remaining disagreements were evaluated by a third reviewer. The entire selection and inclusion process for the final sample of articles took place between August and September 2024.

After the search was conducted, original research that was fully available, featured various methodological designs, had no time restrictions, was published in indexed sources, and was written in English, Portuguese, or Spanish, all of which addressed the established question, was included.

Publications with general descriptions of competency-based education focused solely on conceptual aspects; articles on training for specific intensive care skills (such as ultrasound use or airway management); those on developing competencies through realistic simulation; studies dedicated to specialties such as anesthesiology, surgery, and palliative care; and those targeting undergraduate education were excluded, as they did not address the research question.

### Stage 4: Data analysis and organization

The articles selected for the final sample were fully read by all the researchers independently and individually. The data were subsequently grouped and organized into a table created by the authors to extract information relevant to the study’s development. Two authors tested the table on the first two included articles and then proposed improvements to optimize the extraction of essential study elements and address the guiding question. The following categories were included: author and year, country/region, objective, definitions, professional category and subarea, construction, description, evaluation, and main findings.

As this was a scoping review, no critical appraisal of the included articles was performed, nor was any determination made regarding the quality of the evidence or the results presented in the studies. Nonetheless, the scoping review strategy was the most suitable for this study, as it addressed an emerging topic with the objective of answering a broad question.^(14)^

### Stage 5: Data grouping, synthesis, and presentation

A narrative synthesis of the studies included in the review was conducted, following recommendations described in the international literature.^(16)^ The data from the included articles were explored and compared to identify patterns, which were then grouped by similarity for the extraction and presentation of results. This process was conducted independently by one of the researchers and subsequently validated by the rest of the group.

## RESULTS

**Figure 1:**
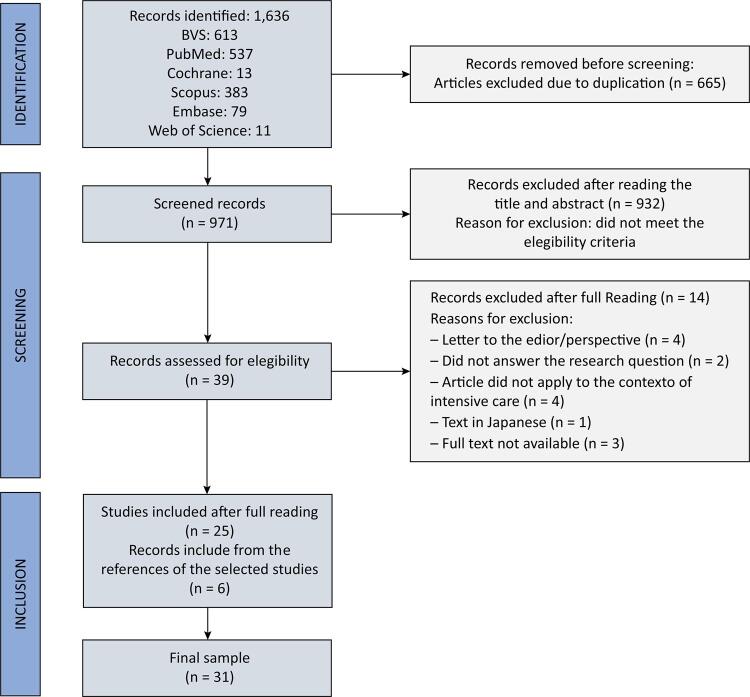
Study process selection.

The initial search identified 1,636 potentially eligible records, and 6 articles were selected from the references, resulting in a final sample of 31 studies included in the review, as described in figure 1. No studies were identified through a gray literature search.

The included articles were published between 2000 and 2024, with most studies focusing on CBE in medical education (n = 20; 64.5%), followed by nursing (n = 10; 32.3%) and physical therapy (n = 1; 3.2%). Most studies were conducted in North America (n = 15; 48.4%), followed by Europe (n = 11; 35.5%), Oceania (n = 3; 9.6%), and Asia (n = 2; 6.5%). The intensive care subspecialties covered in the articles were mixed, with the following subareas identified: adult (n = 22; 70.8%), pediatric (n = 5; 16.2%), neonatal (n = 2; 6.5%), and neurological (n = 2; 6.5%).

The conceptual strategies of CBE identified in the studies were heterogeneous and varied over time. There was an initial focus on the description of skill lists or inventories (n = 3; 10%)^(17-21)^ in the early 2000s, followed by domains and competency statements (n = 9; 29%) in the mid-2010s.^(22-26)^ This progressed to the incorporation of core competencies (n = 8; 26%) and milestones and EPAs (n = 6; 19%) from 2014,^(27-34)^ reflecting an evolution in strategies for competency development and assessment.

Eighteen studies (58%) addressed aspects related to the development of CBE, whereas 13 (42%) presented the outcomes of its implementation. Thus, the findings of this scoping review ranged from studies covering the process of developing CBE (construction, validation techniques, consensus, description, and content) to works discussing the impacts of its implementation on curricula and training programs, as well as on student assessment within the context of CBE.

Considering the narrative synthesis,^(16)^ the results of this review were categorized and presented under three themes: (1) development of CBE; (2) implementation of CBE in curricula, continuing education and training programs; and (3) student assessment.

Table 1 presents the study results according to thematic grouping, detailing the author and year of publication, country/region, intensive care subarea/professional category, and conceptual strategies of each article. The studies are identified as E1 through E31, in chronological order of publication.

**Table 1:** Characterization of results according to thematic grouping, author and year of publication, country/region, intensive care subarea/professional category, and conceptual strategies

	Author, year	Country/region	Subarea/professional category	Conceptual strategies
Studies on CBE development	Dunn et al., 2000^(^^17^^)^	Australia	Adult/Nursing	Competency standards and domains
	Bench et al., 2003^(^^18^^)^	England	Adult/Nursing	Competency statements
	Scholes et al., 2003^(^^19^^)^	England	Adult/Nursing	Competency domains
	CoBaTrICE Collaboration, 2006^(^^20^^)^	United Kingdom	Adult/Medicine	Domains and competency statements (CoBaTrICE)
	Mayer et al., 2006^(^^21^^)^	USA	Neurology/Medicine	Skill list
	Marshall et al., 2007^(^^22^^)^	Australia	Adult/Nursing	Learning outcome statements.
	Buckley et al., 2009^(^^23^^)^	USA	Adult Medicine	Specific and essential competencies (ACGME)
	Sweeney et al., 2009^(^^24^^)^	USA	Neonatology/Physiotherapy	Clinical competencies
	Lovegrove et al., 2012^(^^25^^)^	United Kingdom	Mixed/Nursing	Skill inventory
	Long et al., 2013^(^^26^^)^	Australia and New Zealand	Pediatric/Nursing	Competency standards
	Fessler et al., 2014 E19^(^^27^^)^	USA	Adult/Medicine	EPAs and Milestones
	Hu et al., 2016^(^^28^^)^	China	Adult/Medicine	Essential competences
	Kopf et al., 2018^(^^29^^)^	USA	Adult/Nursing	Essential competences
	Heath et al., 2020^(^^30^^)^	USA	Adult/Medicine	Milestones
	Cui et al., 2020^(^^31^^)^	China	Neurology/Medicine	Essential competences
	Turner et al., 2020^(^^32^^)^	USA	Pediatric/Medicine	Task list
	Werho et al., 2022^(^^33^^)^	USA	Pediatric/Medicine	EPAs and Milestones
	Chlebowskiet al. 2024^(^^34^^)^	USA	Pediatric/Medicine	EPAs and Milestones
Implementation of CBE in curricula, continuing education and training programs	Wilde et al., 2009^(^^35^^)^	United Kingdom	Adult/Medicine	Domains and competency statements (CoBaTrICE)
	Chudgar et al., 2009^(^^36^^)^	USA	Adult/Medicine	Essential competencies (ACGME)
	Bion et al., 2011^(^^37^^)^	United Kingdom	Adult/Medicine	Domains and competency statements (CoBaTrICE)
	Hatfield et al., 2012^(^^38^^)^	United Kingdom	Mixed/Nursing	Skill inventory
	Castellanos-Ortega et al., 2014^(^^39^^)^	Europe	Adult/Medicine	Domains and competency statements (CoBaTrICE)
	Castellanos-Ortega et al., 2021^(^^40^^)^	Spain	Adult/Medicine	Domains and competency statements (CoBaTrICE)
Student assessment	McGaughey, 2004^(^^41^^)^	England	Adult/Nursing	Clinical competencies
	McLean et al., 2005^(^^42^^)^	United Kingdom	Adult/Nursing	Competency statements
	Clay et al., 2007^(^^43^^)^	USA	Adult/Medicine	Essential competencies (ACGME)
	Carey et al., 2013^(^^44^^)^	USA	Neonatal/Medicine	Essential competencies (ACGME)
	McCallister et al., 2015^(^^45^^)^	USA	Adult/Medicine	Essential competencies (ACGME)
	Emke et al., 2019^(^^46^^)^	USA	Pediatric/Medicine	EPAs
	Heath et al., 2021^(^^47^^)^	USA	Adult/Medicine	Milestones

CBE - competency-based education; CoBaTrICE - competency-based training in intensive care medicine in Europe; ACGME - Accreditation Council for Graduate Medical Education in the United States; EPA - entrustable professional activities.

### Topic 1: Development of competency-based education

The process of developing the CBE conceptual strategies identified in the studies was primarily conducted using mixed consensus techniques (n = 12; 39%), with a predominance of the nominal group and the Delphi technique or its modified version.^(20, 22-24,27-34)^

In the selected studies, the identification, construction, and validation of CBE typically followed three stages:

**Stage 1 - Idea generation:** This stage involved collecting suggestions for competencies from various health care professionals, educators in the field, members of specialist societies^(19-25,27-34)^ and, in some cases, patients and their families.^(20)^Methods reported in this stage included the use of online questionnaires and forms, as well as in-person or remote discussion groups. Three studies^(17,18,26)^mentioned the use of practice analysis and observation as methods to identify predominant professional activities, which were then described as competencies.

**Stage 2: Structuring and classification:** The suggestions collected in Stage 1 were analyzed, edited, and grouped into categories or domains. Each competency was subsequently ranked by its importance and the level of autonomy/independence required at different stages of training.^(20,22-24,27-34)^

**Stage 3: Review and refinement:** The results from Stage 2 were reviewed and analyzed in greater depth by a panel of experts, with the feedback received used to refine the list of competencies, ensuring their applicability and relevance. ^(20,23,28-34)^

The content covered in the description of competencies can be grouped into six main categories: (1) management of acute diseases and conditions;^(20,28,29,31)^(2) therapeutic interventions and system/organ support;^(20,28,31)^(3) practical procedures;^(20,23,28,31)^(4) professionalism, ethics, and communication;^(17,20,28,29)^ (5) patient safety and quality management;^(20,28,31)^ and (6) research and education.^(28,29,31)^

The development of CBE ranged from local studies,^(17,21,22,24-26)^to predominantly national definitions of competency standards.^(18-20,23,27-34)^On the one hand, adopting national parameters promotes uniformity in training, facilitates the redeployment of qualified personnel in resource-scarce scenarios such as pandemics, and simplifies national certification processes.^(20)^ On the other hand, establishing regional criteria enables more accurate evaluations that better reflect local clinical practices.^(22)^ The authors also highlight the need to review and update competency descriptions as clinical practice evolves, ensuring that they reflect contemporary health care practices and incorporate new technologies and procedures that may become standards of care while phasing out those that may become obsolete.^(30,38)^

Regarding the length of competency lists, two studies^(30,46)^noted the risk of overly extensive lists with excessive detail, which could lead to reading fatigue and inattentiveness, contributing to their trivialization and improper completion. However, the authors also emphasize that excessively broad and generic lists could compromise the evaluation process, creating insecurity among evaluators and resulting in imprecise feedback.^(30,46)^

### Topic 2: Implementation of competency-based education in curricula, continuing education and training programs

Competency lists serve as guides for the development of training curricula, prominently including programs based on the Competency-Based Training in Intensive Care Medicine in Europe (CoBaTrICE) framework^(35,37,39,40)^and those grounded in the core competencies proposed by the Accreditation Council for Graduate Medical Education (ACGME) in the United States.^(36,43-45,47)^

These studies highlight that the implementation of CBE in curricula facilitates the evaluation of the quality of training programs.^(35)^One study noted that assessments began to rely on measurable criteria for the quality of student experiences and training outcomes rather than being exclusively based on infrastructure parameters, such as the number of beds or admission rates.^(37)^Two studies^(39,40)^ highlighted that the national implementation of competency-based curricula can serve as a benchmark for other services and residency programs. Study E28^(47)^ reports the opportunity to track historical and current data for each program, demonstrating the progression of competencies over time and their variation for each trainee within a year. This allows for identifying potential evaluation biases among supervisors. This approach enables the periodic evaluation and review of training programs to foster improvements in curricula, assessment methods and tutor development.^(47)^

The training of tutors is discussed as one of the main challenges in implementing CBE.^(35,41)^Low reliability in competency assessments, a lack of preparation and specific training in education, insufficient time dedicated to teaching, resistance to change, and the absence of criteria for appointing evaluators are cited as obstacles.^(19,35,41)^To address this gap, studies suggest strategies including protected time for teaching, differentiated remuneration, and the development of programs that focus on training feedback techniques.^(35,36,41)^

Other challenges reported on the implementation of CBE in curricula and training programs include the demand for time, human, and financial resources, particularly for the deployment of assessment technologies, such as electronic portfolios.^(36,39,40)^Additionally, institutions may face difficulties in allocating dedicated time for teaching and evaluation, especially in environments with high workloads and staff shortages.^(36,39,40)^

The development of CBE facilitated the implementation of competency-based training and continuing education programs, particularly in nursing and physical therapy.^(17-19,22,24,29)^ In studies E1,^(17)^ E2,^(18)^ E3,^(19)^ E8,^(22)^ E15,^(25)^ E16,^(38)^ and E17,^(26)^ the competency model was used for the training and integration of nurses already practicing or newly introduced to clinical practice. This approach aimed to address gaps and overcome potential heterogeneities in their education, considering the direct entry of novice nurses into intensive care practice.

The involvement of specialist societies in implementing CBE is recognized as a key factor in initiatives to enhance training quality. These societies apply competency standards in their professional certification processes and serve as technical advisors to authorities and regulatory education bodies.^(39,40)^

### Topic 3: Student assessment

**Figure 2:**
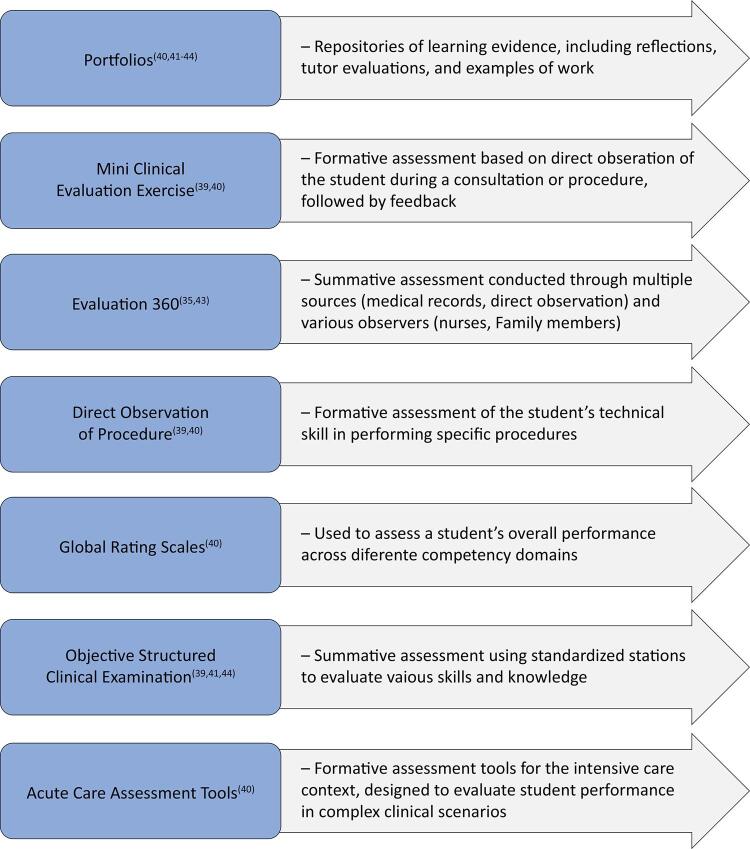
Tools and methods used for student assessment in the context of competency-based education.

Student assessment within the context of CBE, referred to by some authors as workplace-based assessment (WBA),^(43,46)^reveals various tools and methods, which are summarized in figure 2.

The positive aspects of students’ assessment in the CBE context include a more transparent learning process, with clear expectations for both trainees and assessors,^(40,43,47)^ tracking trainee progress throughout training, enabling the early identification of deficiencies;^(39-43,47)^ and encouraging reflective learning through discussions between students and evaluators.^(40-43,45,47)^

These studies highlight narrative feedback as a key element in training competent learners.^(39-41,43,45)^Constructive and targeted feedback is essential for trainees to understand their strengths and weaknesses, guiding their learning efforts accordingly.^(43)^Moreover, the importance of frequent assessments and a close tutor–resident relationship is emphasized, along with periodic and detailed reviews of achieved and pending objectives. This approach facilitates the development of an individualized plan tailored to the specific needs of each trainee.^(40-43)^

Only one study^(44)^ reported on care outcomes following the implementation of CBE in a neonatal intensive care medicine program. Another study highlighted that the quality of the training environment and the care context significantly impact how well skills are applied in real-world situations.^(35)^

Article E9^(43)^identifies challenges in trainees’ assessment within the CBE context, particularly the difficulty of implementing effective, robust longitudinal assessment systems that monitor progress at various training stages, which may require investment in infrastructure and technology. It also highlights challenges with conducting accurate workplace assessments due to factors such as time constraints and busy clinical environments, which can affect the completeness and reliability of evaluations, even when simplified tools are employed.^(36,43)^The lack of a unified assessment model and the limitations of certain evaluative methods can undermine the implementation of CBE, leading to imprecise evaluations with low interrater reliability and difficulties in reducing subjective biases.^(38,42)^Furthermore, the use of unclear and complex language in competency descriptions has been recognized as a significant obstacle.^(30,46)^

Addressing these barriers requires essential involvement from academia to promote research in education and support the development of reliable, replicable, and valid assessment tools.^(41)^Strategies for implementing competency-based assessments include the use of multiple assessment methods that consider various competency domains, encouraging the active participation of residents in their learning process, adopting digital technologies with platforms that facilitate data collection, and considering the opinions of tutors and residents about assessment methods.^(39-47)^

## DISCUSSION

Most of the studies were conducted in North America and Europe. This can be attributed to the significant attention given by the North American medical community to competency-based curricula, particularly following the publication of the milestones by the ACGME.^(48)^ In Europe, the adoption of CBE was strongly driven by the CoBaTrICE framework, which has been implemented in 15 countries across the continent.^(40)^Additionally, other authors highlight the predominance of the English language in publications on CBE.^(49,50)^

Most of the studies included in this review focused on medical education. Regarding the implementation of CBE in other health professions, studies have been conducted in the fields of nursing,^(51)^ veterinary medicine,^(52)^ physiotherapy,^(53)^ pharmacy^(54)^and dentistry.^(55)^ However, these are primarily conducted on the undergraduate education level and are fewer in number than are publications in the medical field.^(56)^ The limited number of publications on milestones and EPAs for other health professions may be explained by the fact that this is an area under development within the medical field itself. Advances in medical CBE may serve as catalysts for similar progress in other health professions.

The studies on CBE in nursing and physical therapy included in the sample focused on the training and continuing education of professionals who are already practicing or have been recently introduced to clinical practice. This may be because these professionals often enter intensive care practice directly, without a gradual integration process, such as that provided by residency programs. These programs typically offer a training period with onsite support.^(57)^ As a result, these professionals are expected to master all activities, from the simplest to the most complex, within a short time frame.^(57)^ Therefore, the development of CBE in these professional categories is seen as a strategy to address potential gaps in training, particularly in critical care.^(57)^

For the conceptual strategies of CBE identified in the included studies, the observed heterogeneity may reflect the challenge in defining competency. In this context, although the concepts of competencies, learning objectives, EPAs and milestones are distinct, the process of describing and constructing these strategies in practice is complex.^(58)^This is related to the fact that competencies are not merely attributes of the individual—such as their knowledge, skills, and attitudes—but rather a product of their interaction with clinical practice, requiring a context in which they manifest and can be evaluated.^(58,59)^ Thus, these constructs are attributes of the individual, which are inherently present but invisible until they are expressed through the execution of an activity, task, or responsibility.^(59)^

These findings highlights the multifaceted nature of competencies, which encompass dimensions of education, training, and practice.^(60)^While they describe individual attributes, the complete description of competencies for a specific specialty can also serve to define the scope of professional practice; serve as a reference for professional certification processes; guide curriculum development and the design of training programs; enable the evaluation of professionals already practicing or newly entering clinical practice; and facilitate the assessment of trainees.^(60)^

As identified in this review, other authors highlight the trend of evolving from the adoption of essential skills and competency lists to the concepts of milestones and EPAs as a way to operationalize CBE.^(5)^ Strategies for describing essential competencies tend to produce extensive lists of the required knowledge, skills, and attitudes. These lists are often broad, with general descriptions that are challenging to evaluate in the context of clinical practice. Moreover, they fail to depict learner progression, making it difficult to implement them in educational and assessment strategies.^(61)^ Thus, the implementation of CBE through milestones and EPAs appears to be a viable alternative, as it enables outcome-based assessment. This approach allows verification of results through the completion of day-to-day tasks, as performing an activity requires the integration of various competencies.^(61)^

In the studies included in the sample, the use of EPAs remains limited; EPAs were not utilized as an evaluative and formative end goal but rather as a reference for the activities that trainees must master by the end of their training. In this context, it is argued that the primary contribution of EPAs has been to enable more robust assessments of trainees. When introducing the EPA concept, Ten Cate^(62)^ emphasized the importance of conducting entrustment decision-making. The author explained that, while efforts are made to establish objective and measurable criteria for trainee evaluation, a portion of the judgment inevitably involves subjective impressions and analyses.^(63,64)^Trusting someone requires a calculated estimation of the risk involved in allowing the trainee to perform the activity unsupervised, weighing the likelihood of undesirable events and their capacity to manage unfamiliar situations.^(63,64)^

To ensure this approach, five levels of supervision are proposed, through which the trainee progressively gains autonomy and independence, with the required supervision level gradually decreasing. This contributes to the decision to allow safe, unsupervised practice.^(63,64)^The five levels of supervision are (1) observation only and (2) direct supervision with the supervisor present onsite. The trainee may perform the activity jointly with the supervisor or independently under direct and reactive supervision and may be ready to intervene if necessary. (3) The third level is indirect supervision, where the supervisor is not present onsite but is readily and quickly available for reactive or on-demand supervision. The supervisor may review all findings or only the most relevant findings. (4) The fourth level is limited supervision, where the supervisor is not physically present but can be reached by phone if needed or is unavailable but provides retrospective feedback and monitoring. (5) Finally, the fifth level acts as a supervisor for the EPA for beginners.^(63,64)^

Thus, in entrustment decision-making, the patient is brought to the center of the evaluation, enhancing patient safety. Trusting someone requires more than an average of past performance; it is forward-looking trust based on future estimations, making it a prospective evaluation.^(63,64)^

Few studies have focused on the structural and cultural organizations necessary for CBE implementation or on faculty training. Furthermore, the evaluation of the positive aspects of CBE in the studies included in the sample was mostly qualitative, describing the perceptions of tutors, program directors, and trainees. Quantitative data are lacking, particularly for assessing the impacts of CBE implementation on quality of care and patient safety. Therefore, the assessment of the impact and effectiveness of CBE implementation should progress to align with Level 4 of Kirkpatrick’s evaluation framework.^(65)^Thus, the evaluation process progresses from reaction and learning to behavior changes, ultimately leading to the results, which may include assessments of quality-of-care indicators and patient satisfaction.^(65)^

The selected studies focused on presenting the development of CBE, with a smaller percentage addressing strategies and outcomes of its implementation. It becomes evident that merely describing competencies does not guarantee changes in training and assessment processes. Competencies outline what is expected. However, their implementation requires profound changes in the teaching–learning process, involving cultural shifts in how evaluations are traditionally conducted, as well as the willingness and commitment of all stakeholders: health care professionals, tutors, students, academics, and specialist societies.

**Figure 3:**
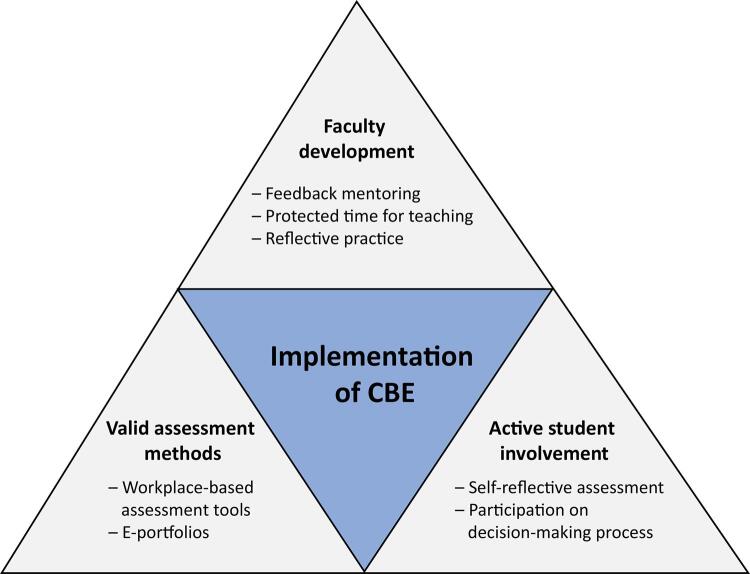
Key elements for the implementation of competency-based education.

Therefore, the successful implementation of CBE relies on the interdependent triangulation of three key elements: faculty development, the integration of effective assessment tools, and active student participation in the teaching–learning process (Figure 3).

CBE - Competency-based education.

Although the training of tutors may impose a challenge, some authors have proposed guidelines for implementing faculty development, which includes 4 dimensions: (1) observation and workplace assessment with the use of simulation and standardized students; (2) feedback and coaching skills with a focus on the SMART technique and narrative feedback; (3) self-assessment and reflective practice during dialog sessions involving trainees and faculty; and (4) attention to building a community of practice among participants.^(66)^

For assessment tools and student engagement, the use of electronic portfolios enables the tracking of students’ progress through different assessment methods and encourages self-reflection among residents. This approach provides a more accurate, transparent, and clear evaluation, as it includes feedback from different assessors at various times while the student completes different tasks.^(67)^ This approach can also help reduce unconscious bias in the evaluation process by involving different assessors with diverse perspectives, ensuring that the evaluation is fair and accurate.^(67)^ An Indonesian study^(67)^ revealed that it is possible to implement app-based e-portfolios in resource-limited scenarios to support CBE implementation. App-based e-portfolios help in the transition from paper to digital, which could reduce costs with material printing, optimize educators’ time, and reduce the amount of physical storage needed.^(67)^ Additionally, mobile apps can make the e-portfolio available to many users, even in places with poor internet access, since data can be synced whenever a connection is available.^(67)^

There were fewer articles on the development and implementation of CBE in nursing and physical therapy. Additionally, we found no studies published in Latin America, underscoring the importance of promoting research on CBE in the context of multiprofessional training for intensive care in resource-limited regions and other areas of health care. Studies on CBE implementation in this context should involve the participation of all stakeholders, including academic institutions and professional societies.

The studies reviewed had several limitations, including a lack of quantitative data on the effects of CBE implementation, inconsistent methods for evaluating competencies, a predominance of qualitative research, and publication bias. Additionally, the diverse methodologies used in the studies made it difficult to compare and synthesize the results, which affects the ability to generalize the findings.

Nevertheless, given that this is a complex, multifaceted, and underexplored topic in intensive care, the inclusion of studies with diverse approaches allowed for broader discussions, including challenges and practical implications for integrating CBE strategies into training curricula.

## CONCLUSION

The development of competency-based education has occurred primarily through consensus techniques, notably the nominal group and the Delphi technique. A transition was observed from initial conceptual strategies, such as skill lists and competency domains, to more robust approaches, including milestones and entrustable professional activities.

The studies highlighted the positive impact of competency-based education on curriculum design, the quality of training programs, and tracking trainee progress, promoting a more transparent and student-centered learning process.

However, challenges persist, such as the need for faculty training, resistance to change, and difficulties in securing time and resources for the effective implementation of competency-based education. Potential strategies include the development of tutors with protected time and differentiated remuneration, encouragement of self-assessment and reflection with active student involvement in the teaching–learning process, and the creation of valid and reliable assessment instruments.

Future research to evaluate the impact of competency-based education on care quality and patient safety, as well as its implementation in Latin American countries and other health professions, is crucial to consolidating this approach. Such efforts are essential to ensure the training of competent professionals who are prepared to face the challenges of clinical practice in intensive care settings.
